# Aluminothermic Synthesis of Dispersed Electrides Based on Mayenite: XRD and EPR Study

**DOI:** 10.3390/ma15248988

**Published:** 2022-12-16

**Authors:** Alexander M. Volodin, Roman M. Kenzhin, Aleksandr V. Kapishnikov, Andrey Y. Komarovskikh, Aleksey A. Vedyagin

**Affiliations:** 1 Boreskov Institute of Catalysis, 630090 Novosibirsk, Russia; 2 Department of Natural Sciences, Novosibirsk State University, 630090 Novosibirsk, Russia; 3 Nikolaev Institute of Inorganic Chemistry of SB RAS, 630090 Novosibirsk, Russia

**Keywords:** calcium aluminate, mayenite structure, aluminothermic reduction, XRD, EPR

## Abstract

The evolution of the structure and the phase composition of a dispersed mayenite at its interaction with metallic aluminum was studied in a temperature range from 900 to 1400 °C in both argon and air atmospheres. The aluminum loading was varied from 0 to 50 wt%. It was found that the addition of aluminum significantly affects the stability of the mayenite and other calcium aluminate phases within the studied temperature range. The formation of the electride state registered by the appearance of a characteristic electron paramagnetic resonance (EPR) signal from F^+^-like centers (g~1.994) in an argon atmosphere was shown to take place already at 1150 °C due to an aluminothermic reduction of this material. The super-narrow (H_p-p_ < 0.5 G) EPR spectra from F^+^-like centers, which were recently observed for the core–shell structures of the C12A7@C type only, were registered for mayenite for the first time. The results obtained in the present study testify firstly towards the possibility of significantly diminishing the temperatures required for the formation of the electride state in such systems and secondly towards the ability to stabilize the size of small electride nanoparticles within the synthesized calcium aluminate matrix.

## 1. Introduction

Among the most interesting and exciting functional materials discovered during the last 20 years, inorganic electrides based on calcium aluminates with a mayenite structure should be especially mentioned. The possibility of their synthesis was demonstrated by Prof. Hosono and co-authors for the first time [1,2,3]. It is worth noting that mayenite itself (12CaO·7Al_2_O_3_, usually labeled as C12A7) has been known for a long time. It was widely applied as one of the components of particular types of cement [4,5,6] and had attracted interest due to the ability to easily change its properties by varying the exact composition of an anion extraframework. It was confirmed that such variations do not lead to any changes in the mayenite structure. Its cationic framework [Ca_24_Al_28_O_64_]^4+^ remains unchanged in all the cases. The composition of the unit cell of such a compound can be described by the following formula: 1 unit cell = [Ca_24_Al_28_O_64_]^4+^∙4X^−^, where 4X^−^ is anion compensating the positive charge of cation. Various negatively charged ions (O^−^, O_2_^−^, O^2–^, OH^−^, Cl^−^, and F^−^) can serve as such anions [7,8,9,10]. These anions are highly mobile within the stable cation framework and, therefore, can be substituted even at moderate temperatures below 1000 °C [10,11]. Prof. Hosono and co-authors have discovered the ability to form the state of electride in these structures when electrons play the role of anions compensating the framework’s charge (i.e., 4X^−^ = 4e^−^). This discovery motivated some research on the properties of this class of materials and a search for areas of its possible application [12,13,14,15,16,17,18,19,20,21,22]. Thus, one of the prospective directions in applying such materials is their use as catalysts [23,24,25,26,27,28,29]. As is known, there are many requirements for catalytic systems, and dispersity is one of the main parameters within these requirements. Exactly the value of the specific surface area of the material defines predominantly its applicability to be used as a catalyst or an adsorbent [30,31]. It is quite obvious that the preparation method, used in the pioneer works [1,2,3] to synthesize the C12A7:e^−^ electrides at high temperatures from the melt, did not allow their obtaining in the dispersed form. On the other hand, a number of methodological approaches have already been developed to produce dispersed mayenites of such compositions as C12A7:O^2−^ and C12A7:OH^−^, and others [29,32,33,34,35,36,37,38]. Evidently, the further calcination of these materials at elevated temperatures in a vacuum or a medium of any inert gas in order to form the electride state has led to a sharp decrease in specific surface area. Such a behavior is typical for calcium aluminates, in general [39]. For this reason, various groups of researchers have made significant efforts to develop methods to obtain the electride state in this material at lower temperatures, allowing it to maintain a sufficiently high specific surface area. The most effective way to achieve this has been to use metallic calcium and aluminum as reagents. These metals are able to initiate the processes of calciumthermic [1,40,41] and aluminothermic [41,42,43,44] reduction of mayenite during its synthesis due to the high formation heat values for corresponding oxides.

Another approach to obtaining dispersed electrides proposed in our recent works [45,46,47] is associated with the processes of carbothermic reduction of mayenite nanoparticles inside the carbon shell. In such cases, the deposited carbon coating prevents direct contact with the initial mayenite particles and their sintering at high temperatures. At the same time, the presence of carbon initiates the processes of carbothermic reduction of mayenite and allows the formation of the electride state within the core–shell structure (C12A7@C) at moderate temperatures below 1250 °C.

It should be mentioned that, in the majority of the cited papers [1,40,41,42,43], metallic aluminum and calcium serving as reducing agents were added into the initial mixture of reagents taking into account the final stoichiometry corresponding to the composition 12CaO∙7Al_2_O_3_. Contrarily, the processes of a carbothermal reduction described in works [45,46,47] consider the interaction of the reductant (carbon, in this case) with the already formed mayenite structure due to the elimination of oxygen ions, which does not enter the cationic framework:

C12A7:O^2−^ + C → C12A7:e^−^ + CO(CO_2_) (1)

As shown in many papers published previously [3,12,40,42], the formation of the electride state in mayenite is always accompanied by the appearance of a typical singlet EPR signal (g~1.994; H_p-p_~5–7 G) from F^+^-like centers. The concentration of such centers does not exceed 2·10^19^ g^−1^, which correlates well with the conductivity of the samples and reflects the substitution degree of oxygen anions with electrons [3]. Therefore, this feature can be applied to diagnose the formation of the electride state in the mayenite samples under study.

The present work aimed to study the conditions for the aluminothermic reduction of mayenite by a similar mechanism due to the interaction of metallic Al with the already-formed structure of mayenite. Such experiments are reported for the first time. Of particular interest for possible catalytic applications of synthesized materials was the behavior of such systems with a significant excess of the metal introduced, when—along with mayenite—other aluminum-containing oxide phases are expected to appear. A series of samples with varied aluminum content was synthesized and characterized by low-temperature argon adsorption/desorption, electron paramagnetic resonance, and X-ray diffraction analysis.

## 2. Materials and Methods

### 2.1. Synthesis of the Mayenite Samples

The mayenite samples studied in the present work were prepared as described elsewhere [32,46,48]. Aluminum hydroxide (pseudo-boehmite, Pural SB-1, Condea Chemie GmbH, Hamburg, Germany) and calcium carbonate (special purity, Reachim, Moscow, Russia) were used as starting materials without any purification. The first stage was the decomposition of CaCO_3_ with the formation of CaO in a muffle at 950 °C in the air for 6 h. Then, the resulting CaO was added at continuous stirring to a suspension of aluminum hydroxide in distilled water at room temperature. The final ratio of the components corresponded to the mayenite stoichiometry (12CaO∙7Al_2_O_3_). The mixture thus obtained was thoroughly stirred in distilled water for 10 h, filtered, dried at 110 °C, and calcined in a muffle at 500 °C in the air for 6 h. The obtained C12A7 sample (labeled as CA-500) was used as a starting material for further synthesis. This sample is mostly represented by the mayenite phase [32,48].

The CA-500 sample was mixed with fine-dispersed powder of metallic aluminum taken in an appropriate amount and calcined in an argon flow (or in air, if specified). The obtained samples were denoted as CA-T-Al(X), where T is the calcination temperature and X is the content (wt%) of aluminum introduced. The samples calcined in the air were named similarly to CA-T-Al(X)-O_2_. In all the cases, the heating was performed with a temperature ramping rate of 3 °C/min and with maintaining at the final temperature point (T) for 6 h.

### 2.2. Characterization of the Samples

The values of specific surface area (SSA) were determined by the Brunauer–Emmett–Teller (BET) method. The data were obtained by low-temperature argon adsorption using an ASAP-2400 analyzer (Micromeritics Instrument Corp., Norcross, GA, USA).

X-ray powder diffraction (XRD) analysis was made on an ARL X’tra diffractometer (ThermoFisher Scientific, Waltham, MA, USA) with a Cu–K_α_ radiation source (λ = 1.5418 Å). The diffraction patterns were registered in a 2θ range of 15–85° with a step of 0.05° and a signal accumulation time of three s per step. Rietveld profile refinement of the XRD patterns was calculated using a GSAS–II program [49].

The Electron Paramagnetic Resonance (EPR) spectra were recorded at room temperature using a Varian E-109 spectrometer (Varian Instruments, Palo Alto, CA, USA) operating in the X–band. The *g*-factors were obtained with reference to a standard 2.2-diphenylpicrylhydrazyl (DPPH) resonance at *g* = 2.0036. The weighted portion of copper (II) sulfate pentahydrate (CuSO_4_·5H_2_O) was used to evaluate the concentration of paramagnetic species. The intensities of EPR spectra were determined by numerical double integration with baseline compensation using standard OriginPro software (v. 9.1.0; OriginLab Corp., Northampton, MA, USA).

## 3. Results and Discussion

### 3.1. Characterization of the Initial CA-500 Sample

During this study, a series of synthesis experiments with a variation in the concentration of aluminum added and the calcination temperature of the samples in an argon atmosphere were carried out. The XRD pattern of the starting mayenite used as a precursor is shown in Figure S1. It should be noted that the initial system, along with the mayenite phase, contains an impurity phase of calcium oxide and, possibly, X-ray amorphous phases of AlOOH and γ-Al_2_O_3_, which are not observed in the pattern but may present in the system based on the precursor ratio introduced there. The starting material has a sufficiently high dispersity. The values of the coherent-scattering region (crystallite sizes) estimated for mayenite and CaO are about 80 nm, and the specific surface area value can reach 70 m^2^/g. In this regard, the reaction of such a precursor with dispersed metal Al can proceed quite efficiently.

### 3.2. Characterization of the Samples Calcined at 900 °C

**Figure 1:**
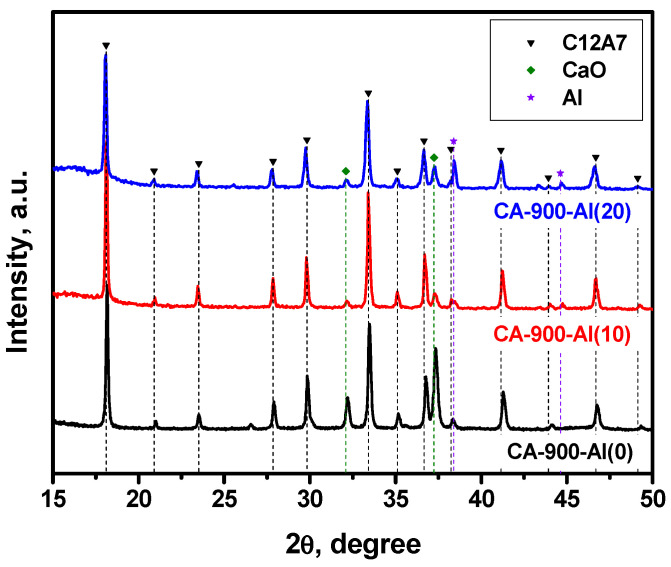
XRD patterns for the mayenite samples with varied content of aluminum additive after calcination in an Ar atmosphere at 900 °C.

The results of the XRD study for the samples containing different amounts of added aluminum calcined at 900 °C in an argon atmosphere are presented in Figure 1 and Table 1. As can be seen from Table 1, the initial CA-900-Al(0) sample calcined at this temperature is dominated by the mayenite phase with a relatively large admixture of unreacted CaO (up to 20%) and X-ray amorphous Al_2_O_3_, the presence of which was not taken into account when calculating the fraction of the phase. When 10 wt% of aluminum was added (CA-900-Al(10) sample), the mayenite phase content is markedly increased and the residual CaO content is reduced. This is quite logical while an excess of CaO can interact with Al to form the mayenite phase. In addition, an impurity of the Al metal phase (~2%) was observed in such a sample, which increased to ~7% in the case of the CA-900-Al(20) sample.

According to the EPR technique, no singlet signal with *g*~1.994 typical for the electride state was observed in the EPR spectra for this series of samples. It can be assumed that the mayenite phase that appeared at 900 °C contains predominantly oxygen anions within the cationic framework, thus forming structures of the [Ca_24_Al_28_O_64_]^2+^:O^2−^ type. It should be emphasized that the lattice parameter for mayenite with additionally introduced aluminum is slightly increased (Table 1).

### 3.3. Characterization of the Samples Calcined at 1150 °C

**Figure 2:**
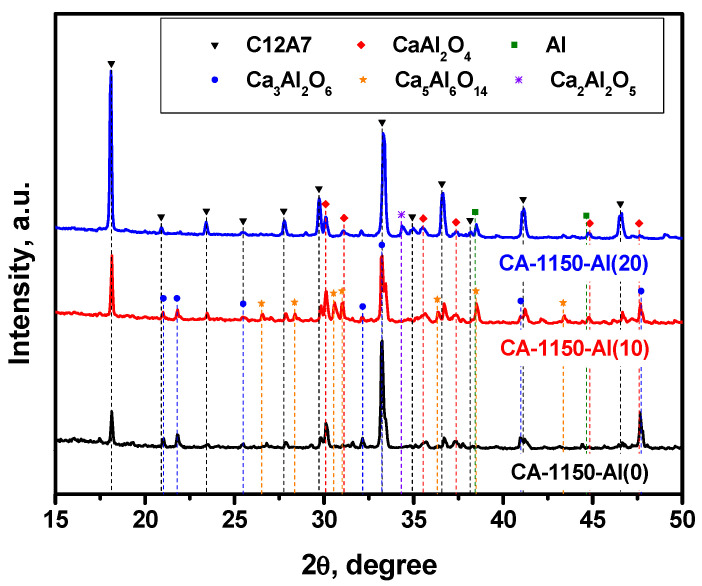
XRD patterns for the mayenite samples with varied content of aluminum additive after calcination in an Ar atmosphere at 1150 °C.

The XRD patterns for a series of samples calcined at 1150 °C are compared in Figure 2. Table 2 shows the calculated lattice parameters along with the phase composition and the SSA values for these samples. It is evidently seen that the calcination of the initial sample at 1150 °C is accompanied by a noticeable decomposition of the mayenite phase and the formation of calcium aluminates of different compositions. These results correlate well with the data recently reported for an analogous system [32]. The addition of 10 wt% Al, CA-1150-Al(10) sample, does not practically affect the concentration of the mayenite phase but influences the ratio of the accompanying phases. Thus, the fraction of the Ca_3_Al_2_O_6_ (C3A) phase decreased by two times, and a comparable amount of the Ca_5_Al_6_O_14_ phase (C5A3) appeared. With an increase in the concentration of added Al to 20 wt%, CA-1150-Al(20), this phase disappears and the relative proportion of mayenite increases by almost three times. It can be supposed that the presence of excess aluminum contributes to the stabilization of the mayenite phase at this temperature. Note also that the fraction of metallic Al phase in these samples is extremely small. However, a further increase in the Al concentration to 50 wt% leads to a significant decrease in the relative proportion of mayenite and a predominance in the phase composition of the metallic Al (Figure S2), which is associated with a shift in the concentration of this chemical element.

**Figure 3:**
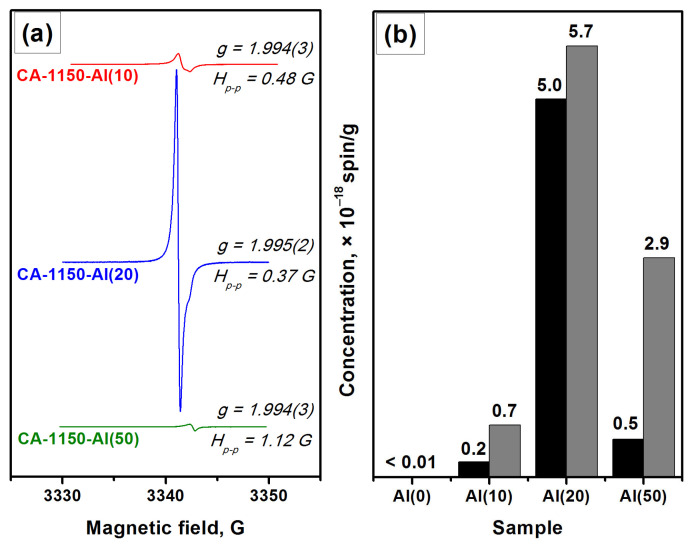
EPR spectra for a series of samples calcined at 1150 °C (**a**) and concentration of F^+^-like centers obtained for these samples (**b**): as-registered (black) and normalized to the content of the mayenite phase (gray).

Characterization by the EPR method shows that for the CA-1150-Al(0) sample, the electride state is not formed and there is no signal from electron centers in the EPR spectra. However, the addition of 10 wt% Al, CA-1150-Al(10) sample, leads to the appearance of an intense EPR signal from F^+^-like centers with a characteristic singlet spectrum with *g*~1.994 (Figure 3). This is an extremely interesting result, given the fact that at this treatment temperature the content of the mayenite phase in this sample does not exceed 30%. Note that for the initial samples without the addition of Al, the formation of the electride state when they are calcined in an argon atmosphere is observed only starting from temperatures of about 1380 °C. Thereby, this temperature can be lowered to 1150 °C by a simple introduction of 10 wt% Al into the initial mayenite. The SSA value for such a sample also increases by almost two times if compared to the CA-1150-Al(0) sample (Table 2). Subsequently, at the same calcination temperature and increasing the aluminum content to 20%, CA-1150-Al(20) sample, the concentration of F^+^-like centers recorded by the EPR method increases by almost an order of magnitude. This can be associated with an increase in the fraction of the mayenite phase in the sample. For the CA-1150-Al(50) sample, the concentration of F^+^-like centers detected by means of EPR drops significantly, which is consistent with the XRD data (Figure S2), indicating a significant decrease in the content of the mayenite phase. It can be assumed that excess aluminum is sintered in an argon atmosphere and contributes to the formation of aluminum-enriched calcium aluminate phases.

**Figure 4:**
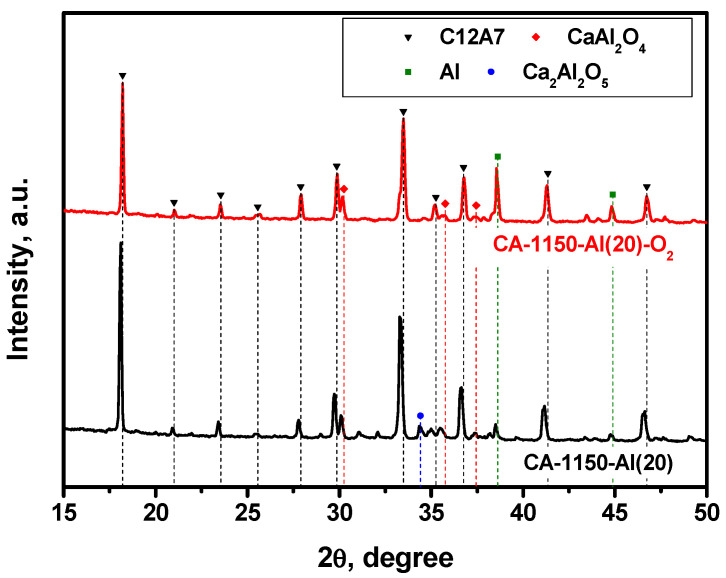
XRD patterns for the CA-1150-Al(20) samples calcined in argon and air at 1150 °C.

In order to compare the effect of the reducing and oxidizing atmosphere on the phase composition of the samples, the mayenite sample containing 20 wt% of aluminum was additionally calcined in air at the same temperature. The XRD patterns for the CA-1150-Al(20) and CA-1150-Al(20)-O_2_ samples are exhibited in Figure 4. By comparing these data, it can be clearly seen that the content of the mayenite phase for CA-1150-Al(20)-O_2_ decreases by almost two times and unreacted metallic aluminum is present in a significant concentration. Most likely, in an oxygen-containing atmosphere, an alumina coating is formed on the surface of aluminum particles, which prevents it from further entering the composition of complex oxides and stabilizes metallic Al in the state of core–shell structures of the Al@Al_2_O_3_ type. The mayenite lattice parameter in such a sample is about 0.03 Å less than that of the CA-1150-Al(20) sample (Table 2). This may be due to the substitution of electrons in the mayenite unit cells by oxygen anions and anion radicals. Note that the EPR signal from F^+^-like centers is also not observed for this sample.

### 3.4. Characterization of the Samples Calcined at 1380 °C

**Figure 5:**
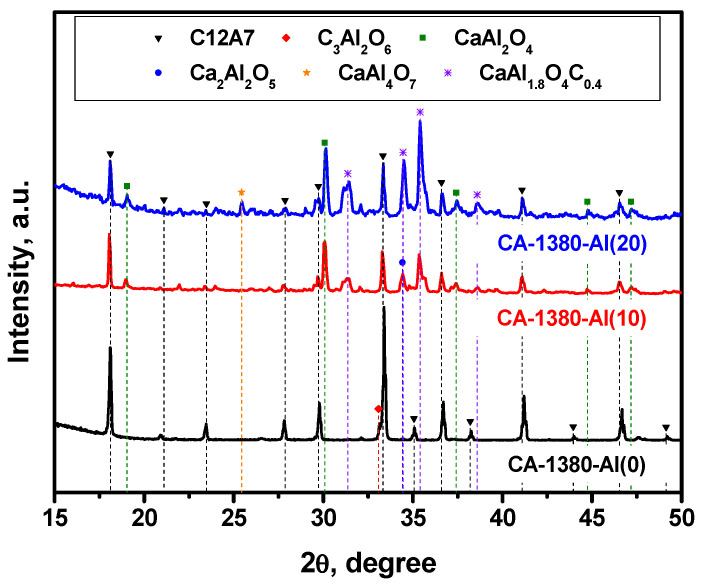
XRD patterns for the mayenite samples with varied content of aluminum additive after calcination in an Ar atmosphere at 1380 °C.

As previously noted, mayenite, when heated in an Ar atmosphere, is able to form the electride state at temperatures above 1380 °C, which is close to its melting temperature. In this regard, it was of particular interest to study the behavior of the material with added metallic aluminum at such a high temperature. The obtained results are summarized in Figure 5 and Table 3.

As it follows from the presented data, the CA-1380-Al(0) sample is essentially represented by single-phase mayenite with a minor admixture of the C3A phase. The addition of Al in an amount of 10 wt% leads to a marked increase in the proportion of the CaAl_2_O_4_ phase that had not been observed before and a decrease in the proportion of mayenite. At the same time, low-intensity reflexes also appear, the estimated contribution of which in the total intensity does not exceed 2–3%, and which can be attributed to the phase of mixed CaAl_1.9_O_4_C_0.4_ oxocarbide. Subsequently, an increase in the concentration of Al from 10 to 20 wt% leads to a decrease in the content of the mayenite phase in the samples (Table 3), the appearance of aluminum-enriched phases CaAl_4_O_7_, Ca_3_Al_10_O_18_, and Al_2_O_3_, as well as an increase in the intensity of the reflexes assigned to CaAl_1.9_O_4_C_0.4_. Since there is no exact structural data for the last phase, it is not possible to quantify its fraction. However, the XRD patterns clearly show that the intensity of mayenite reflexes decreases with an increase in the concentration of added Al, and for the CA-1380-Al(50) sample (Table 3, Figure S3), this phase disappears completely. From the structural data obtained for the CA-1380-Al(20) and CA-1380-Al(50) samples, it can also be concluded that the addition of significant amounts of metallic aluminum followed by calcination of the samples at 1380 °C is accompanied by the formation of a CaAl_1.9_O_4_C_0.4_ phase. Supposedly, carbon appears to be introduced in minor amounts from the graphite crucible used for these syntheses.

**Figure 6:**
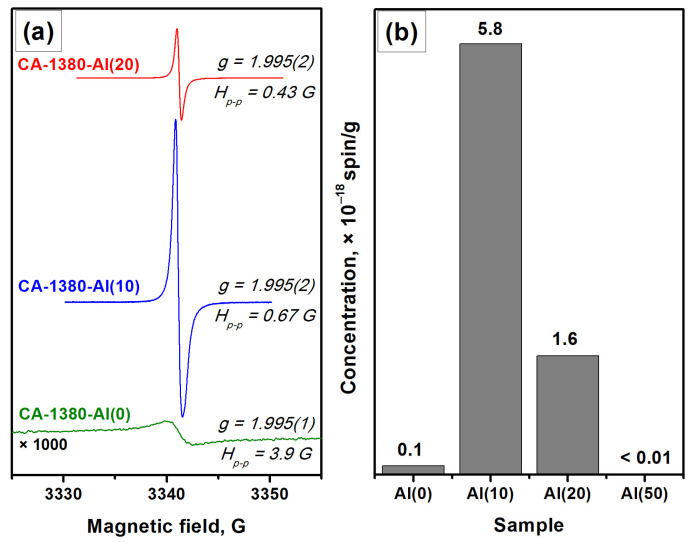
EPR spectra for a series of samples calcined at 1380 °C (**a**) and concentration of F^+^-like centers registered for these samples (**b**).

The EPR data indicate the presence of a signal assigned to F^+^-like centers in the corresponding spectra (Figure 6), thus confirming the formation of the electride state in the studied samples. It is important to note that for these samples (Figure 6) as for the CA-1150-Al(10) and CA-1150-Al(20) samples (Figure 3), the observed EPR signals related to F^+^-like centers are very narrow. Previously, such signals were detected for the electride nanoparticles in the systems of C12A7@C type only [46]. It is assumed that the process of aluminothermic reduction under our conditions may also be accompanied by the appearance of submicron electride particles in the calcium aluminate composite, characterized by this intense and abnormally narrow EPR signal.

It should be noted that an increase in the concentration of added aluminum from 10 to 20 wt% has little effect on the content of the mayenite phase in the samples calcined at 1380 °C. However, it is expected to lead to a noticeable increase in the proportion of aluminum-enriched calcium aluminate phases as well as in the appearance of alumina phases. Finally, during the formation of the CA-1380-Al(50) sample, mayenite is completely decomposed (Table 3, Figure S3) and the EPR signal from F^+^-like centers disappears.

## 4. Conclusions

In the present work, both the evolution of the phase composition and the formation of F^+^-like centers characterizing the appearance of the electride state during the interaction of dispersed mayenite with metallic aluminum in a temperature range from 900 to 1380 °C have been studied. The possibility of forming the mayenite phase in the electride state in such composite systems with a specific surface area of up to 20 m^2^/g in an argon atmosphere at temperatures starting from 1150 °C and concentrations of introduced aluminum up to 20 wt% is demonstrated. The appearance of super-narrow (H_p-p_ < 0.5 G) EPR spectra, previously observed for nanocrystalline electrides in C12A7@C systems only, was discovered for such systems for the first time. This indicates the possible presence of an electride in the form of nanoparticles in the resulting composite material. The addition of a small (~10 wt%) amount of metallic aluminum to mayenite has been shown to stabilize its phase composition near a temperature of 1150 °C when the initial mayenite is largely decomposed. The synthesized composite material with such an excessive amount of aluminum contains a whole set of calcium aluminate phases with a high content of the electride phase and maintains a sufficiently high specific surface area even at a temperature of 1380 °C.

It should also be noted that the reduction in the temperature of the electride state formation in such a system allows not only to obtain this material with a higher specific surface area but also opens up the possibility of using a wider class of possible substrates suitable for producing films of the electride as a material for microelectronics.

## Figures and Tables

**Table 1:** Lattice parameter (*a*), phase composition, and specific surface area (SSA) of the mayenite samples calcined in an Ar atmosphere at 900 °C.

Sample	Phase Composition	*a*, Å	SSA, m^2^∙g^−1^
CA-900-Al(0)	C12A7 (77%) + CaO (23%)	11.967	26
CA-900-Al(10)	C12A7 (95%) + CaO (4%) + Al (1%)	11.989	18
CA-900-Al(20)	C12A7 (87%) + CaO (5%) + Al (8%)	12.001	18

**Table 2:** Lattice parameter (*a*), phase composition, and specific surface area (SSA) of the mayenite samples calcined in an Ar atmosphere at 1150 °C.

Sample	Phase Composition	*a*, Å	SSA, m^2^∙g^−1^
CA-1150-Al(0)	Ca_3_Al_2_O_6_ (52%) + C12A7 (24%) + CaAl_2_O_4_ (24%)	11.984	12
CA-1150-Al(10)	Ca_3_Al_2_O_6_ (24%) + C12A7 (25%) + CaAl_2_O_4_ (23%) + Ca_5_Al_6_O_14_ (26%) + Al (2%)	11.999	20
CA-1150-Al(20)	C12A7 (72%) + Ca_3_Al_2_O_6_ (5%) + CaAl_2_O_4_ (15%) + Ca_2_Al_2_O_5_ (5%) + Al (3%)	12.031	10
CA-1150-Al(20)-O2	C12A7 (41%) + Al_2_O_3_ (9%) + CaAl_2_O_4_ (12%) + Ca_3_Al_2_O_6_ (8%) + Al (30%)	11.996	4.8
CA-1150-Al(50)	C12A7 + Al_2_O_3_ + CaAl_4_O_7_ + CaAl_2_O_4_ + Al	11.981	4

**Table 3:** Lattice parameter (*a*), phase composition, and specific surface area (SSA) of the mayenite samples calcined in an Ar atmosphere at 1380 °C.

Sample	Phase Composition	*a*, Å	SSA, m^2^∙g^−1^
CA-1380-Al(0)	C12A7 (93%) + Ca_3_Al_2_O_6_ (7%)	11.991	<1
CA-1380-Al(10)	CaAl_2_O_4_ (64%) + C12A7 (30%) + Ca_2_Al_2_O_5_ (6%)	12.025	4.4
CA-1380-Al(20)	C12A7 + CaAl_2_O_4_ + Al_2_O_3_ + Ca_3_Al_10_O_18_ + CaAl_4_O_7_ + CaAl_1.9_O_4_C_0.4_	11.989	-
CA-1380-Al(50)	CaAl_1.9_O_4_C_0.4_ + CaAl_4_O_7_ + Al_2_O_2_C + Al	-	6.5

## Data Availability

Data is contained within the article.

## Supplementary Materials

The following supporting information can be downloaded at: https://www.mdpi.com/article/10.3390/ma15248988/s1, Figure S1: XRD pattern for the initial mayenite samples (CA-500); Figure S2: XRD patterns for the CA-1150-Al(50) sample calcined in Ar at 1150 °C; Figure S3: XRD patterns for the CA-1380-Al(50) sample calcined in Ar at 1380 °C.
