# Supplementary material for: Aluminothermic Synthesis of Dispersed Electrides Based on Mayenite: XRD and EPR Study
Source: Materials (Basel). 2022 Dec 16;15(24):8988. doi: 10.3390/ma15248988 (PMC9781158; doi:10.3390/ma15248988)
Supplement: Supplementary file 1 [file materials-15-08988-s001.zip › materials-2084307-supplementary.pdf]

materials

Supplementary Information

Aluminothermic Synthesis of Dispersed Electrides Based on Mayenite: XRD and EPR Study

Alexander M. Volodin [1,*], Roman M. Kenzhin [1,2], Aleksandr V. Kapishnikov [1,2], Andrey Yu. Komarovskikh [3] and Aleksey A. Vedyagin [1]

[1] Boreskov Institute of Catalysis, 630090 Novosibirsk, Russia
2 Department of Natural Sciences, Novosibirsk State University, 630090 Novosibirsk, Russia
[3] Nikolaev Institute of Inorganic Chemistry, 630090 Novosibirsk, Russia
* Correspondence: volodin@catalysis.ru (A.M.V.)

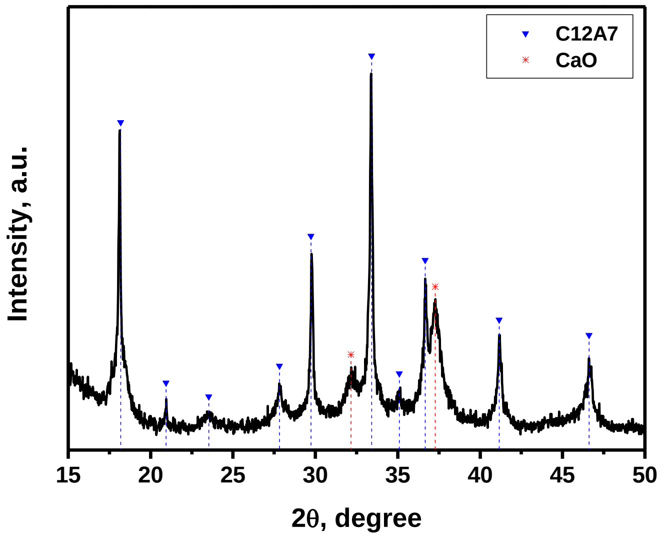

Figure S1. XRD pattern for the initial mayenite samples (CA-500).

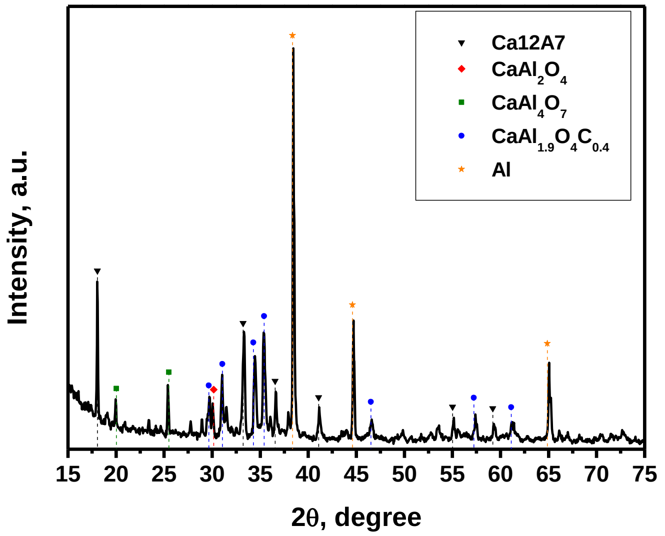

Figure S2. XRD patterns for the CA-1150-Al(50) sample calcined in Ar at 1150 °C.

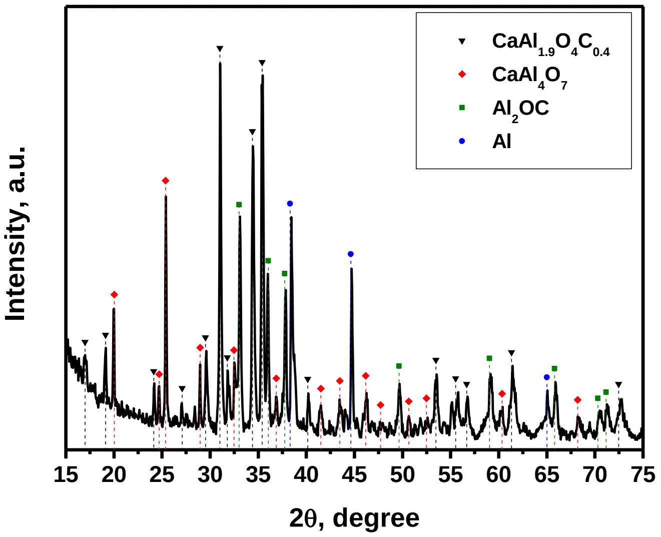

Figure S3. XRD patterns for the CA-1380-Al(50) sample calcined in Ar at 1380 °C.
